# Statistical and Methodological Inconsistencies Regarding a Cross‐Sectional Study Conducted Towards Hepatitis B Post‐Exposure Prophylaxis in Ethiopia

**DOI:** 10.1002/hsr2.73114

**Published:** 2026-08-26

**Authors:** Muhammad Umair Ahmad, Muhammad Rayyan Humayun, Mohammad Abbas Khan, Tahoor Shahzad

**Affiliations:** ^1^ Department of Medicine, Dow Medical College Dow University of Health Sciences Karachi Sindh Pakistan

Dear Editor,

Given the significance of post‐exposure prophylaxis (PEP) in developing countries like Ethiopia, the efforts of Esubalew Muluneh Aligaz et al. [1] in their research titled *“Knowledge, Attitudes, Practices, and Associated Factors of Healthcare Workers Toward Hepatitis B Post‐Exposure Prophylaxis in Ethiopia: A Cross‐Sectional Study”* cannot be commended enough. Although the study addresses relevant issues related to PEP, we feel that there are inconsistencies in the paper that call the legitimacy of the findings into question.

We found that Table 3 contains blatant mathematical and data validity issues. Several categories show sample sizes exceeding the initially stated number of 400, or upon calculation, yield significant changes. In addition to this, the values are laden with typing errors; for example, the “!” in AOR of 4–6 years of service. Moreover, many reported crude and adjusted odds ratios fall outside their stated ranges, with no justification, including mathematically impossible negative odds ratios.

The study document reported that 77.8% of healthcare workers failed to use PEP, but it offers no explanation for this issue. Without the use of an established behavioural framework like the Health Belief Model [2], the study cannot interrogate the causes of non‐use, like risk perception or institutional barriers. Adapted tools require demonstrated construct validity beyond internal consistency alone.

Section 4.4 and Table 4 contain multiple contradictions, most notably that the authors state that only 16.5% trust PEP effectiveness, directly contradicting Table 4, which shows 48% agreed it prevents infection. Furthermore, item frequencies mistakenly total to 450 instead of *N* = 400. Lastly, the claimed 70% positive attitude rate lacks verifiable aggregate data. Such inconsistencies may compromise the reliability of the findings [3].

We feel that, due to the aforementioned limitations and inherent flaws in the methodology of the study, the findings must be interpreted with caution. Larger and well‐conducted studies would be required in order to reach a more well‐informed consensus regarding the impact of the many factors affecting Hepatitis B PEP in developing countries.

## Author Contributions

**Muhammad Umair Ahmad:** writing – original draft, writing – review and editing. **Muhammad Rayyan Humayun:** writing – original draft, writing – review and editing, supervision. **Mohammad Abbas Khan:** writing – original draft, writing – review and editing. **Tahoor Shahzad:** writing – original draft, writing – review and editing.

## Funding

The authors nothing to report.

## Conflicts of Interest

The authors declare no conflicts of interest.

## Data Availability

Data sharing not applicable to this article as no datasets were generated or analysed during the current study.
